# Pre-adapted Arch Bar Revisited for Open Reduction and Internal Fixation in Mandibular Fractures at Tooth-Bearing Sites

**DOI:** 10.5812/traumamon.18169

**Published:** 2014-03-24

**Authors:** Kazuhiko Yamamoto, Yumiko Matsusue, Satoshi Horita, Tadaaki Kirita

**Affiliations:** 1 Department of Oral and Maxillofacial Surgery, Nara Medical University, Kashihara, Japan

**Keywords:** Arch bar, Fracture, Mandible

*Dear Editor,*

Mandibular fractures are frequently encountered in oral and maxillofacial surgery. Open reduction and internal fixation (ORIF) is one of the treatments of choice (1-3). Maxillomandibular fixation (MMF) is usually performed during surgery (2-5). Recently, an arch bar was reconsidered as a means of providing additional stability to internal fixation in anterior mandibular fractures (1, 5). The arch bar needs to be secured to the dental arch spanning the fracture line at the same time as anatomical reduction and achieving proper occlusion. Here, we present a method of open reduction and internal fixation (ORIF) using a pre-adapted arch bar for intraoperative MMF in mandibular fractures at tooth-bearing sites.

An impression is taken of both mandibular and maxillary dental arches and plaster models are fabricated. Two models are made for the mandible. One is sectioned at the fracture line into two segments to make a reduced mandibular model. The mandibular dental arch of the sectioned model is precisely reduced to articulate to the maxillary dental arch in reference to the wear facets on the teeth. Then, an arch bar is adapted to the reduced mandibular arch by bending it to make a pre-adapted arch bar. If there are lost teeth within the dental arch, the arch bar can be reinforced to avoid its deformation. Care should be taken since teeth might be displaced due to injury. The other mandibular model is left unsectioned to check the degree of occlusal disturbance. A pre-adapted arch bar is also made for the maxilla. Fracture sites of the mandible are temporally fixed until the operation.

**Figure 1.**
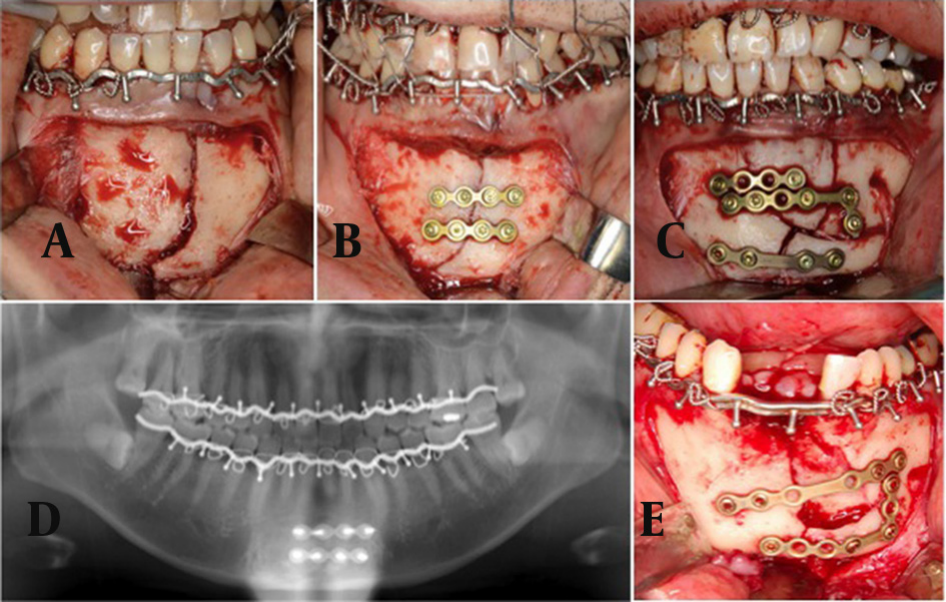
Findings of Representative Cases A: Intraoperative findings of a representative case. A pre-adapted arch bar is secured to the dental arch of the large segment and is loosely secured to that of the small segment. The arch bar is not completely fit to the dental arch of the small segment before reduction, the arch bar is secured to the dental arch of the small segment (B) after reduction. Internal fixation is performed using two miniplates under MMF. In this case, the arch bar is not secured to the anterior teeth at this point and is pulled upward by wiring for MMF, (D) panoramic X-ray findings after surgery. Precise anatomic reduction and proper occlusion were obtained. The arch bar secured is well adapted to the reduced dental arch of the mandible, (C) intraoperative findings of a case of comminuted fracture. An arch bar works as an additional support of the reduced dental arch with comminuted fracture. (E) Intraoperative findings of a case of loss of anterior teeth. An arch bar is reinforced in the area of lost teeth.

In ORIF, a pre-adapted arch bar is firstly secured to the maxillary dental arch. After removal of the temporary fixation, the pre-adapted arch bar is secured to the dental arch in the large segment of the mandible with 0.5 mm wires. The ends of the wires are cut to an appropriate length and twisted. Across the fracture line, the arch bar is placed along the dental arch in the small segment and is loosely secured to the teeth with wires. The arch bar does not completely fit the dental arch of the small segment before reduction (Figure 1). Then, an incision is made in the vestibular mucosa and the fracture line is exposed. Bone fragments are held with forceps and the fracture is manually reduced. After confirmation of precise anatomical reduction and proper occlusion, the arch bar is completely secured to the dental arch of the small segment by tightening the wires. Since premolar and molar teeth do not have basal tubercles, holding the wires under the tubercles by an instrument is not necessary during wire tightening. Then, maxillomandibular fixation (MMF) is performed with three wires in the anterior and molar regions. Internal fixation is principally performed using two miniplates in the symphyseal region or one miniplate in the region posterior to the mental foramen along Champy’s ideal osteosynthesis line against tensile and torsional forces (5, 6). After the release of MMF, occlusion and mandibular movement is checked. The ends of the wires are cut at an appropriate length and twisted. The wound is closed by sutures. Since incisors are weaker than molars, fixation with wires is sometimes avoided or performed after miniplate fixation with 0.4 mm wires so as not to cause displacement or extrusion of these teeth. If there are any displaced teeth, these can be properly reduced and fixed with the pre-adapted arch bar.

MMF is not necessary after surgery. An X-ray is taken to confirm the status of reduction and fixation. The maxillary arch bar is removed after stable occlusion is confirmed. The mandibular arch bar may be retained for a while, since it works as an additional support against tensile and torsional forces applied under mandibular function, especially in cases with comminuted fracture or conservatively treated condylar fractures, but it is removed within two weeks for gingival health.

ORIF using a pre-adapted arch bar for intraoperative MMF has several advantages for mandibular fractures at tooth-bearing sites as follows:

1. a pre-adapted arch bar works as a guide for the reduced dental arch and can be appropriately secured,
2. it works as an additional support of the reduced dental arch by resisting tensile and torsional forces,
3. is advantageous for the fixation of displaced teeth in proper occlusion.
